# Could Traumatic Brain Injury Be a Risk Factor for Bruxism and Temporomandibular Disorders? A Scoping Review

**DOI:** 10.3390/brainsci15030276

**Published:** 2025-03-05

**Authors:** Beata Pająk-Zielińska, Agnieszka Pająk, Agnieszka Drab, Piotr Gawda, Grzegorz Zieliński

**Affiliations:** 1 Interdisciplinary Scientific Group of Sports Medicine, Department of Sports Medicine, Medical University of Lublin, 20-093 Lublin, Poland; 2 Clinic of Anaesthesiology and Paediatric Intensive Care, Medical University of Lublin, Gebali Str. 6, 20-093 Lublin, Poland; 3 Chair of Preclinical Sciences, Department of Medical Informatics and Statistics, Medical University of Lublin, 20-093 Lublin, Poland; 4 Department of Sports Medicine, Medical University of Lublin, 20-093 Lublin, Poland

**Keywords:** traumatic brain injury, TBI, temporomandibular disorders, TMD, bruxism, sleep bruxism

## Abstract

**Background/Objectives**: Bruxism and temporomandibular disorders (TMDs) are common conditions of the stomatognathic system. Some studies suggest a potential link with traumatic brain injury (TBI), which is gaining increasing interest among researchers. The aim of this scoping review is to map the available evidence on the association between TBI and bruxism or TBI and TMDs. **Methods**: The review was conducted by analyzing four databases: PubMed, Web of Science, Scopus, and the Cochrane Collaboration database. A total of 340 studies were reviewed in this work, and 4 studies examining the connections between TBI were included in the analysis (with n = 3 focusing on the association between bruxism and TBI, and n = 1 on the association between TMDs and TBI). **Results**: Analyzing the publications on bruxism and TMDs in relation to TBI, at this stage it can be concluded that there is no solid evidence confirming the impact of TBI on the studied conditions. This is due to the quantity and quality of the collected evidence. **Conclusions**: In light of the presented review, it must be concluded that the quantity and quality of the evidence are insufficient to assert that TBI is a factor in the onset of bruxism or TMDs. Further research on this phenomenon is recommended, particularly focusing on the effects of different severities of TBI and various regions of brain injury.

## 1. Introduction

The Brain Injury Association of America has proposed the following definition: traumatic brain injury (TBI) is defined as an alteration in brain function or other evidence of brain pathology caused by an external force [1]. The National Institute of Neurological Disorders and Stroke further notes that TBI can result from the head suddenly and forcefully striking an object or when an object penetrates the skull and enters brain tissue [2]. Additionally, the Institute divides the symptoms of TBI, which can range from mild to severe, depending on the extent of the brain injury. In cases of mild TBI, a person may remain conscious or experience a brief loss of consciousness for a few seconds or minutes. Other symptoms of mild TBI include headache, confusion, dizziness, blurred vision or eye strain, ringing in the ears, a bad taste in the mouth, fatigue, changes in sleep patterns, mood swings, or difficulty with memory, concentration, attention, or thinking. A person with moderate to severe TBI may show these same symptoms but may also experience a worsening or persistent headache, repeated nausea or vomiting, seizures, difficulty waking up, dilated pupils in one or both eyes, slurred speech, weakness or numbness in the limbs, lack of coordination, and increased confusion, agitation, or restlessness [2].

In 2020, there were 214,110 TBI-related hospitalizations in the United States, which equals 586 TBI-related hospitalizations per day [3]. Analyzing other statistical data in terms of the incidence of TBI per 100,000 population, in North America there were 1299 cases, in Europe 1012 cases, and the fewest in Africa, with 801 cases [4]. According to the study by Dewan et al., it is estimated that sixty-nine million individuals suffer from TBI every year, regardless of the cause [4].

The incidence of TBI is systematically increasing worldwide, a trend linked to multiple factors such as population aging, greater participation in extreme sports, and a rise in road accidents [5,6,7]. The older population, in particular, faces a higher risk of TBI due to an increased likelihood of falls, often associated with impaired balance, reduced muscle mass, and comorbid conditions [8,9,10]. Beyond immediate treatment and rehabilitation, it is crucial to investigate all potential consequences of TBI [11,12]. Recent reports suggest that TBI may contribute to the development of bruxism or TMDs [13,14].

Given their potential impact on patient functioning, studying the consequences of TBI in the context of TMDs and bruxism is critical [13,14]. Epidemiological studies indicate an increasing incidence of TBI, which can lead to disturbances in the neuromuscular system, raising the risk of developing TMDs and bruxism [3,4]. These conditions, in turn, can result in chronic pain and joint dysfunction, and negatively affect quality of life [15,16,17]. Currently, TMDs and bruxism affect approximately one-third of the global population [18,19], making it essential to identify their potential causes. Hypothetically, TBI could be associated with these disorders.

Bruxism is generally defined by consensus as follows: sleep and awake bruxism are masticatory muscle activities that occur during sleep (characterized as rhythmic or non-rhythmic movements) and wakefulness (characterized by repetitive or sustained tooth contact and/or by bracing or thrusting of the mandible) [20]. The global prevalence of bruxism (both sleep and awake) is estimated at 22.22% (sleep bruxism = 21%, awake bruxism = 23%). However, epidemiological data vary depending on the type of bruxism and the continent. For instance, the prevalence of sleep bruxism was observed at 31% in North America, 23% in South America, 21% in Europe, and 19% in Asia. Awake bruxism showed the highest prevalence in South America, at 30%, followed by Asia at 25% and Europe at 18% [19]. Bruxism occurs on a multifactorial basis [19]. The latest research findings published by Koecklin et al. (2024) suggest that higher brain regions responsible for processing stress and anxiety, as well as nuclei within the trigeminal system, have strong neural connections that likely play a role in the pathophysiology of bruxism. Disruptions in the serotonin and dopamine systems are associated with the development of this disorder, indicating their significant role in its mechanisms [21]. Based on other studies by Pavlou et al. published in the same year, researchers observed that special attention is given to how bruxism impacts the central nervous system, particularly through the activation of the hypothalamic–pituitary–adrenal axis. This results in increased corticosterone levels in the bloodstream, which are also mirrored by elevated salivary cortisol levels, thereby creating a self-sustaining cycle of bruxism [22]. In summary, bruxism may arise as a result of complex interactions between neural systems responsible for emotional, motor, and hormonal responses. These actions, combined with abnormalities in neurotransmission, can lead to a persistent and self-reinforcing cycle in which bruxism is sustained and exacerbated. The 2018 consensus guidelines should also be remembered: “in otherwise healthy individuals, bruxism should not be considered as a disorder, but rather as a behavior that can be a risk (and/or protective) factor for certain clinical consequences” [20].

One of the first works connecting bruxism with brain injury was a case report by Ivanhoe et al. (1999) regarding the treatment of bruxism associated with TBI using botulinum toxin-A [13]. Chaudhuri provides a thorough analysis of hypotheses linking bruxism to TBI. The author suggests that TBI-related damage disrupts multiple central pattern generators (CPGs) in the brain and, in some cases, leads to dysfunctional interactions among neurons within these networks [23].

CPGs can be understood as neural circuits located in the spinal cord or brain that autonomously produce rhythmic movement patterns, such as walking or breathing, without requiring constant input from higher brain regions [24]. Expanding on Chaudhuri’s observations, CPGs typically consist of interconnected neurons that maintain reciprocal functional states. When a part of this network is damaged, the coordinated activity of the CPGs is compromised, resulting in impaired function or “dysfunctional reciprocal relationships” among the neurons. Additionally, the author highlights that CPG dysfunction may be linked to a depletion of available dopamine or a decrease in dopamine receptor density [23]. The above literature demonstrates a multifocal potential influence of TBI on the development of bruxism.

However, the etiology of the occurrence of TBI and temporomandibular disorders (TMDs) is hypothesized to have a different character. The term “TMDs” is used to describe pain and dysfunction in the masticatory muscles and the temporomandibular joint. TMDs are the most common pain-related conditions in the oral and facial regions. The global incidence of TMDs is estimated at 34% [18]. The prevalence of TMDs is significantly higher in South America (47%) compared to Asia (33%) and Europe (29%) [18].

In the comorbidity of TBI and TMDs, the etiopathology can be considered from two perspectives: neurological and biomechanical. From a neurological perspective, TBI and TMDs are associated with activity-dependent synaptic plasticity of projection neurons in the spinal trigeminal nucleus or with central sensitization [25]. The projection neurons of the spinal trigeminal nucleus are responsible for transmitting sensory information, particularly related to pain and touch, from the facial area to the central nervous system, enabling the perception of stimuli from this part of the body [26]. Central sensitization, on the other hand, is a process in which the central nervous system becomes hypersensitive to stimuli, leading to enhanced pain signaling and potentially causing excessive responses to normally mild or harmless stimuli [24]. Given that TMDs and TBI are chronic pain conditions involving the trigeminal nerve and a similar anatomical region, it can be speculated that the presence of central changes associated with TBI may facilitate the occurrence of TMDs [25]. From a biomechanical perspective, consideration should be given to the impact of cranial bone injuries (fractures, cracks) occurring with TBI [27] on changes associated with TMDs [25].

Based on the gathered information, we have decided to conduct further research to analyze the potential impact of TBI on the development of changes in the stomatognathic system. The aim of this scoping review is to map the available evidence on the association between TBI and bruxism or TBI and TMDs, and identify key concepts, methodologies, and research gaps in this area.

## 2. Materials and Methods

This review is following the PRISMA Extension for Scoping Reviews (PRISMA-ScR [28]). The review protocol was registered in the Open Science Framework (OSF) under the number identifier DOI 10.17605/OSF.IO/N83FA.

### The Data Collection Process

The review was conducted by analyzing four databases: PubMed, Web of Science, Scopus, and the Cochrane Collaboration database [11,29]. The review began on 25 October 2024, with a planned completion date of 30 January 2025 [30]. However, due to the limited amount of literature available, the review process was completed on 7 November 2024. Keywords compliant with the MeSH (Medical Subject Headings) database were used for the search (Table 1).

The search methods were based on previous systematic reviews [18,19]. No time restriction was applied for inclusion or exclusion from the study; all studies in the databases were analyzed.

This review was conducted by two independent reviewers (B.P.-Z. and G.Z.) who first assessed the titles of the papers, followed by the abstracts, and finally the full papers. All disputes were resolved by P.G. [18,19].

Exclusion Criteria: study design, narrative reviews, systematic articles, meta-analyses, expert opinions, case reports or series of patients, and studies in languages other than English. Inclusion criteria, based on PICO standards (P—population, I—intervention, C—comparison, O—outcome), are presented in Table 2 [31].

A complete synthesis of the results is conducted in a descriptive form in the Results section.

## 3. Results

**Figure 1:**
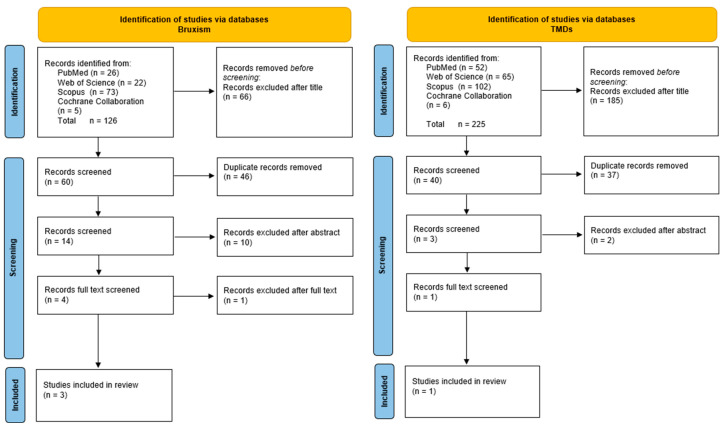
PRISMA flow diagram.

Based on the criteria described above, 126 studies related to bruxism and TBI were identified. After reviewing the titles, 66 studies were excluded, leaving 60 to be screened. Duplicates were removed, and abstracts were assessed, after which full-text evaluations were conducted (at this stage, one study was excluded [32]). Ultimately, three studies were included in the analysis (Figure 1).

For studies concerning TMDs and TBI, 225 records were identified, of which 185 were excluded after title evaluation. This left 40 studies, from which 36 duplicates were removed. The remaining studies were assessed based on their abstracts, with two being excluded. One study then qualified for full-text evaluation and was subsequently included in this review (Figure 1).

The presentation of results is divided into subsections. Details of the qualified scientific papers can be found in Table 3.

There are many uncertainties regarding the existence of a strong connection between TBI and bruxism or TBI and TMDs. Although some studies suggest a possible association between these issues, the results are inconsistent and often contradictory. Chaudhuri [23] and Kothari et al. [33] describe possible connections between TBI and bruxism. However, these studies were conducted on small sample sizes without control groups. In contrast, the study by Suzuki et al. [34] (Table 3) does not support these findings. The lack of conclusive results in this area may suggest that bruxism could be linked to other factors not necessarily related to TBI.

Similarly, while TBI may lead to certain changes in the nervous system that could theoretically influence the occurrence of TMDs, there is no definitive evidence to suggest that brain injuries are the primary cause of these disorders. One study (Karpuz et al. [35]) was found, but changes in pain processing may also occur in contexts unrelated to head trauma, which further complicates efforts to identify the cause of TMDs in patients with TBI.

Therefore, despite some suggestions, the lack of conclusive evidence means that the association between bruxism, TMDs, and brain injuries remains largely unconfirmed (Table 3). The limited amount of available literature indicates a significant gap in research in this area.

**Table 3:** Details of qualified scientific papers.

Author	Group Details	Diagnosis of TBI	Diagnosis of the Stomatognathic System	Suggested Mechanism
Bruxism				
Chaudhuri [23]	12 participants (2 females, 10 males) Age range: 3 to 58 years	The author describes the use of the Glasgow Coma Scale (GCS) [36]. However, the description in Table 1 in [23] suggests the use of imaging studies.	In this study, bruxism was examined based on clinical observations and physical symptoms, such as teeth grinding and masseter muscle spasms, which were visible in all patients.	The main mechanism linking TBI and bruxism is the damage to CPGs in the brainstem, which are responsible for controlling rhythmic jaw movements. TBI may lead to dysfunction of these structures and disturbances in the dopaminergic system, resulting in uncontrolled clenching and teeth grinding. Pramipexole, as a dopamine agonist, may help normalize these processes, thereby reducing the symptoms of bruxism.
Kothari et al. [33]	10 participants, 4 classified as TBI, (1 female, 4 males) Age range: 20 to 63 years	The author describes the use of the Early Functional Ability [37] and Ranchos Los Amigos Scale [38], but no other studies were mentioned. The patients were treated at the Neurorehabilitation and University Research Clinic, Hammel, Denmark, which suggests additional studies related to TBI that are not described in the paper.	An electromyographic study recorded EMG activity of the anterior temporalis muscles for two hours in two different sessions, in patients in a state most similar to sleep. The EMG device detected “bruxism-like behavior” [39] using a moving average algorithm, analyzing episodes of muscle activity exceeding three times the background level [40].	TBI can lead to bruxism through disturbances in the functioning of the central pattern generator in the brainstem and dysfunction of the autonomic nervous system, resulting in increased jaw muscle activity. This may serve a compensatory function, helping to maintain airway patency and mucosal hydration. However, it also leads to negative outcomes, such as excessive tooth wear and muscle overload, and its treatment in TBI patients is particularly challenging.
Suzuki et al. [34]	n = 24 with mTBI (9 females, 15 males), control group n = 20 (12 female, 8 men) Mean age of mTBI group: 38 ± 11, mean age of control group: 31 ± 9	The confirmation of TBI was based on an examination by a neurosurgeon, who conducted an assessment according to the diagnosis criteria of the World Health Organization Task Force [41]. The diagnosis included a GCS score of 13–15 at the time of medical care admission. The period of loss of consciousness, mental disorientation, and/or post-traumatic amnesia did not exceed 30 min after the injury.	A polysomnographic study assessed sleep stages based on 20-s epochs of electroencephalography (EEG), electrooculography (EOG), and EMG of the mentalis muscle, following the modified Rechtschaffen and Kales criteria [42]. Sleep parameters such as total sleep time, sleep efficiency, REM sleep latency (time from lights off to the onset of REM sleep), micro-arousals (MAI—micro-arousal index), apnea–hypopnea index (AHI, frequency of apnea and hypopnea episodes), and periodic limb movement index (PLMI, frequency of involuntary limb movements) were analyzed. The frequency of rhythmic masticatory muscle activity (RMMA) during sleep was assessed based on EMG recordings of the masseter muscle and video recordings, classifying episodes as phasic (a series of at least three bursts within 2 s), tonic (a sustained contraction lasting more than 2 s), or mixed. Additionally, the amplitude and frequency of RMMA episodes were calculated relative to total sleep time.	Current findings suggest that the occurrence of mild traumatic brain injury (mTBI) is not associated with an increased risk of bruxism. However, in patients with mTBI who experience persistent and difficult-to-manage headaches, it is advisable to conduct a diagnostic evaluation for bruxism, as it may influence their symptoms.
Temporomandibular Disorders				
Karpuz et al. [35]	30 patients with TBI (10 females, 10 males) and 30 in the control group, both women and men Mean age of control group: 35.33 ± 9.6, mean age of TBI group: 37.47 ± 9.295	A physical examination for the presence of complications related to TBI.	The examination for TMDs was conducted using the Fonseca Questionnaire [43] for temporomandibular joint dysfunction, the range of motion of the temporomandibular joint, the mandibular angle, and the pain pressure threshold of the temporalis and masseter muscles.	TBI leads to TMDs through the activation of neuronal mechanisms, such as central sensitization and synaptic plasticity within the trigeminal system. Damage to neural structures, including the facial and trigeminal nerves, can cause sensory disturbances, muscular asymmetry, and chronic pain through excessive activation of microglia and increased pain conduction in the central nervous system. Additionally, alterations in pain processing within the brainstem and sensory cortex may exacerbate TMD symptoms, which explains the high prevalence of this condition in TBI patients, particularly those with post-traumatic headaches.

## 4. Discussion

This review responds to the call by Babiloni et al., who encouraged research on the impact of TBI on TMDs [25]. The aim of this scoping review is to map the available evidence on the association between TBI and bruxism or TBI and TMDs, identify key concepts, methodologies, and research gaps in this area.

Traumatic brain injury (TBI) could potentially contribute to the development of bruxism through various neuroanatomical and neurophysiological pathways. Damage to CPGs, which regulate rhythmic motor activities, may disrupt the coordination of jaw movements, leading to involuntary parafunctional activity. Additionally, TBI often results in imbalances in dopamine and serotonin systems, impairing motor control and reducing inhibitory regulation over masticatory muscles [21,23,44].

TBI-related disruptions in brain regions responsible for stress and emotional processing, such as the prefrontal cortex, amygdala, and hypothalamus, can further increase the risk of bruxism. Strong neural connections between these areas and the trigeminal system suggest that heightened stress responses may lead to excessive jaw muscle activity [21,45].

Moreover, TBI can dysregulate the hypothalamic–pituitary–adrenal axis, increasing cortisol levels and creating a self-sustaining stress cycle. Elevated stress hormones reinforce bruxism, which in turn perpetuates neuromuscular dysfunction [22].

In summary, the hypothetical explanation for the possible influence of TBI on the development of bruxism suggests that TBI may cause damage to motor control networks, neurotransmitter systems, and stress regulation pathways, which could collectively contribute to bruxism. These mechanisms interact in a feedback loop, where disrupted neuronal functions and heightened stress responses sustain and exacerbate the condition over time. However, it is important to emphasize that these are hypothetical foundations. The studies are not conclusive on this matter. The most recent research identified only two studies suggesting a connection, with one study examining mild TBI providing a counterpoint (Table 3). This highlights a clear research gap and the difficulties in gathering appropriate study groups. Different brain regions are associated with TBI-related damage, and there are different levels of TBI itself. Therefore, future research may need to focus on specific regions and levels of damage related to TBI and their consequences, rather than analyzing global TBI.

This complexity is mirrored in other factors that influence the temporomandibular joint and related masticatory muscles. For instance, Sharma et al. identified factors such as behavioral, social, emotional, and cognitive components [46]. Additionally, recent studies have observed that factors like wearing masks [47], having visual impairments [48,49], and injuries such as whiplash [50,51] also affect masticatory muscles.

Despite the potential connections presented in the introduction, which could hypothetically explain the associations between TBI and bruxism or TMDs, no significant evidence was found in the current literature. During the literature review phase, a notable number of case studies were identified. For example, Kesikburun et al. described the case of a man with TBI who complained of severe nighttime teeth grinding, which began two months after the injury [52]. Another instance linking bruxism and TBI was described by Yi et al. [53].

Regarding the connection between TMDs and TBI, Meng et al. described two cases of intra-articular ankylosis of the temporomandibular joints following TBI [14]. In the present review, only one study was identified, including 30 patients with traumatic brain injury (TBI) and 30 individuals in the control group [35].

As previously hypothesized, the coexistence of TBI and TMDs can be examined from two perspectives: neurological and biomechanical. TBI may lead to the activation of neuronal mechanisms, such as central sensitization and synaptic plasticity within the trigeminal system. Damage to neural structures, including the facial and trigeminal nerves, may result in sensory disturbances, muscular asymmetry, and chronic pain due to excessive microglial activation and increased pain transmission in the central nervous system [26].

From a neurological perspective, head trauma and TMDs are associated with synaptic plasticity dependent on the activity of projection neurons in the spinal trigeminal nucleus, which are responsible for transmitting sensory information—particularly pain and touch—from the facial region to the central nervous system. Central sensitization, defined as an increased sensitivity of the central nervous system to stimuli, leads to enhanced pain signaling and may cause exaggerated responses to normally mild stimuli. Head trauma-related damage may contribute to the development of TMDs, particularly through alterations in pain processing within the brainstem and sensory cortex, thereby exacerbating TMD symptoms [25,26].

From a biomechanical perspective, cranial bone injuries, such as fractures or fissures, also play a significant role in the development of TMD-related changes. The high prevalence of TMDs among patients with head injuries, especially those experiencing post-traumatic headaches, suggests a strong association between these two conditions [26].

However, current evidence does not confirm a direct link between TBI and TMDs, as only one relevant publication has been identified. Despite the theoretical foundations and a single study emphasizing the impact of TBI on TMDs, this evidence remains insufficient. Additionally, it is important to note that TMDs is a general term encompassing various disorders affecting the masticatory muscles, the temporomandibular joint, and surrounding tissues [18,54]. Future studies should differentiate the effects of TBI on the development of specific TMD subtypes. Similarly, research on the relationship between TBI and the onset of bruxism remains an area requiring further investigation.

When analyzing this study, it is important to emphasize that it has several limitations, primarily the limited amount of current literature investigating the potential impact of TBI on bruxism or TMDs. This highlights a research gap that warrants further scientific investigation.

Future research on TBI and its impact on bruxism and TMDs should focus on several key aspects. It is essential to investigate both the biological mechanisms and the clinical consequences of TBI.

Future studies should differentiate the severity of TBI and its potential impact on TMDs and bruxism, analyzing how specific severity levels influence different types of TMDs. For instance, research should determine whether TBI is more frequently associated with the muscular form of TMDs.

A similar approach should be applied to bruxism research. While bruxism is most commonly reported when patients are unaware of the behavior, such as during sleep, future studies should also examine the relationship between TBI and awake bruxism.

## 5. Conclusions

In light of the review presented, it can be concluded that the current evidence, in terms of both quantity and quality, is insufficient to establish a definitive link between traumatic brain injury and the onset of bruxism or temporomandibular disorders. Given these limitations, further research is crucial, particularly focusing on the impact of varying severities of TBI and the effects of injury in different regions of the brain. These areas warrant more investigation to better understand the potential association between TBI and bruxism or temporomandibular disorders.

## Figures and Tables

**Table 1:** Research finding strategy.

Databases	PubMed	Web of Science	Scopus	Cochrane Collaboration
Keyword compliant	“Traumatic brain injury” AND ‘’bruxism” “Brain injury” AND “bruxism” “TBI” AND “bruxism” “traumatic brain injury” AND “temporomandibular disorders” “Brain injury” AND “temporomandibular disorders” “TBI” AND “temporomandibular disorders”	“Traumatic brain Injury” AND “bruxism” “Brain injury” AND “bruxism” “TBI” AND “bruxism” “traumatic brain injury” AND “temporomandibular disorders” “Brain injury” AND “temporomandibular disorders” “TBI” AND “temporomandibular disorders”	“Traumatic brain injury” AND “bruxism” “Brain injury” AND “bruxism” “TBI” AND “bruxism” “traumatic brain injury” AND “temporomandibular disorders” “Brain injury” AND “temporomandibular disorders” “TBI” AND “temporomandibular disorders”	“Traumatic brain injury” AND “bruxism” “Brain injury” AND “bruxism” “TBI” AND “bruxism” “traumatic brain injury” AND “temporomandibular disorders” “Brain injury” AND “temporomandibular disorders” “TBI” AND “temporomandibular disorders”

**Table 2:** PICO summary of inclusion criteria.

	Inclusion Criteria
Population	
	Individuals who have suffered a traumatic brain injury (TBI), including both adults and children, regardless of the severity of the injury. This group may include patients in clinical settings, rehabilitation centers, or emergency departments.
Intervention	
	The evaluation of the incidence and severity of bruxism and temporomandibular disorders (TMDs) in individuals with a history of TBI. This may involve diagnostic assessments, questionnaires, and clinical evaluations to identify and measure the presence of these conditions.
Comparison	
	The comparison group could consist of individuals without a history of TBI, matched for age, gender, and other relevant factors. This group would serve as a baseline to assess the differences in the prevalence and severity of bruxism and TMDs.
Outcome	
	The primary outcomes of interest would be the incidence rates of bruxism and TMDs among individuals with TBI compared to those without. Secondary outcomes could include the severity of symptoms, quality-of-life assessments, and any potential correlations between the severity of the brain injury and the severity of bruxism or TMD symptoms.

## Data Availability

Not applicable.
